# The Antibacterial Effects of Apacaries Gel on *Streptococcus mutans*: An *in vitro* Study

**DOI:** 10.5005/jp-journals-10005-1241

**Published:** 2014-08-29

**Authors:** Apa Juntavee, Jomjai Peerapattana, Ariya Ratanathongkam, Nartsajee Nualkaew, Supaporn Chatchiwiwattana, Panta Treesuwan

**Affiliations:** Associate Professor, Department of Pediatric Dentistry, Faculty of Dentistry, Khon Kaen University, Khon Kaen, Thailand; Associate Professor, Department of Pharmaceutical Technology, Faculty of Pharmaceutical Sciences, Khon Kaen University, Khon Kaen Thailand; Associate Professor, Department of Oral Biology, Faculty of Dentistry, Khon Kaen University, Khon Kaen, Thailand; Lecturer, Department of Pharmacognosy and Toxicity, Faculty of Pharmaceutical Sciences, Khon Kaen University, Khon Kaen Thailand; Associate Professor, Department of Oral Biology, Faculty of Dentistry, Khon Kaen University, Khon Kaen, Thailand; Graduate Student, Department of Pediatric Dentistry, Faculty of Dentistry, Khon Kaen University, Khon Kaen, Thailand

**Keywords:** Antibacterials, Streptococcus mutans, Apacaries gel

## Abstract

**Background:** New approaches for chemomechanical caries removal require effective materials with antibacterial properties for removal of infected dentin. Apacaries gel is a newly developed material comprised polyphenol from mangosteen extracts and papain mixed in gel preparation.

**Aim:** This study evaluated the antibacterial effects of Apacaries gel on *Streptococcus mutans in vitro.*

**Materials and methods:** Mangosteen pericarp powder was extracted. The amount of phenolic compounds was determined using the Folin-Ciocalteu method. The time-kill kinetics were investigated. Mangosteen extract and papain were mixed with gel base to develop Apacaries gel. The inhibition zone of the Apacaries gel was determined using agar well diffusion methods.

**Results:** The mangosteen pericarp extract, which contains α-mangostin, was active against *S. mutans* strain ATCC25175. The time-kill kinetics curve showed that applying 1 mg/ml of mangosteen extract can reduce *S. mutans* by 50% within approximately 5 seconds; after this reduction, the bacterial count rapidly dropped to 0 within 60 seconds. Using mangosteen extract and papain mixture gel preparation resulted in a larger inhibition zone than using the mangosteen extract gel or papain gel separately.

**Conclusion:** Apacaries gel can effectively inhibit *S. mutans* strain ATCC25175. Apacaries is capable of *S. mutans* inhibition better than both mangosteen extract or papain separately.

**How to cite this article:** Juntavee A, Peerapattana J, Ratanathongkam A, Nualkaew N, Chatchiwiwattana S, Treesuwan P.

The Antibacterial Effects of Apacaries Gel on *Streptococcus mutans:* An *in vitro* Study. Int J Clin Pediatr Dent 2014;7(2):77-81.

## INTRODUCTION

In young children, the risk of caries has been shown to be greater when the colonization of *Streptococcus* (*S. mutans*) in the mouth occurs earlier.^1^ Dental caries is a major cause of pain and infection, which can have severe consequences for the quality of life of the affected children and their families.^2^ Avoiding the early colonization of *S. mutans* may lead to favorable long-term effects on the caries experience and the need for restorative treatment.^3^ A previous approach to the treatment of caries using hand excavation was a painful, ineffective and tedious method for caries removal.^4^ Rotary instruments from low speed to ultrahigh speed evolved from that approach. Thermal and pressure effects on the pulp produced pain and were major drawbacks to rotary instruments.^5^ Due to the shortcomings of the drill, alternative techniques, such as air abrasion, ultrasonic instrumentation, lasers and a chemomechanical approach to caries removal, such as Carisolv and Papacaries materials were developed.^6^ Out of these techniques, air abrasion, sonoabrasion, ultrasonic instrumentation and lasers are costly and tooth-sensitive methods and therefore less frequently used.^4^

The chemomechanical approach is the most documented alternative to traditional drilling.^7^ Chemomechanical caries removal (CMCR) involves the chemical softening of a carious dentin, followed by its removal with gentle excavation. Apacaries gel is a novel dental material and composed of a mixture of polyphenol from mangosteen extracts and papain in a gel preparation. This gel was developed for caries removal with gentle excavation in primary teeth. There are several *in vitro* studies investigating the effect of specific polyphenols against *S. mutans*. Some of the studies report the inhibition of glucosyltransferase (GTF) dependent insoluble glucan synthesis.^8,9^ Some studies report the inhibition of acid production by *S. mutans* and partly ascribe this result to the inhibition of the proton translocating bacterial enzyme F-ATPase.^10-13^ F-ATPase transports protons out of cells and alleviates the negative influence of acidification on metabolic processes, thus, decreasing the pH of the extracellular environmental.^14^ One study reports the inhibition of mutans adherence to hydroxyapatite.^15^ ‘Papain is an enzyme extracted from the latex of the leaves and fruit of the adult green papaya, *Carica papaya*. This enzyme is an endoprotein similar to human pepsin, which has bactericidal, bacteriostatic and anti-inflammatory activity, and is a debriding agent. Papain does not damage healthy tissue. In contrast, it accelerates the cicatricial process and has bacteriostatic and bactericidal action. Papain acts by cleaving the collagen molecules that are partially destroyed by the action of caries and can digest dead cells and eliminate the fibrin coat formed by the caries process’.^16^ In addition, papain acts only on carious tissue, which lacks the plasmatic protease inhibitor alpha-1 antitrypsin, but its proteolytic action is inhibited on healthy tissue, which contains alpha-1 antitrypsin.^17^ Apacaries gel is composed of papain and polyphenol from mangosteen extracts; therefore, the hypothesis of this study was focused on antibacterial effects of this novel dental material.

## AIM

The purpose of this study is to evaluate the antibacterial effects of Apacaries gel against *S. mutans in vitro*.

## MATERIALS AND METHODS

### Preparation of mangosteen Crude Extract

Mangosteen pericarp powder was purchased from^4^ Spectrum, USA. The mangosteen powder (200 gm) was macerated in 95% ethanol at 25°C for 3 days with continuous shaking and then filtered under vacuum. The extract was evaporated using a rotary evaporator and freeze dryer. The crude extract was kept at –20°C until use. The chemical pattern of the crude extract was determined using thin layer chromatography (TLC) as described by Pothitirat and Gritsanapan.^18^ Briefly, TLC (Silica gel 60 F_254_, Merck, Darmstadt, Germany) was used with the mobile phase of CHCl_3_-EtOAc-MeOH (8:1:0.5), sprayed with 10% H_2_SO_4_ in ethanol and heated at 110°C for 10 minutes. Then, the compounds were observed under UV light of 366 nm. Standard α-mangostin (Biopurify, Chengdu, China) was used as reference compound.

### Determination of Total Phenolic Content

The total phenolic content of the mangosteen extract is given as milligram gallic acid equivalents per 100 mg extract sample (mg GAE/100 mg extracts). It was determined using a Folin-Ciocalteu reagent^19^ in a 96-well plate. The 200 μl reaction mixtures contained 20 μl of diluted samples, 100 μl of 10% Folin-Ciocalteu reagent, and 80 μl of 7% Na_2_CO_3_. After 30 minutes of incubation at room temperature, the absorbance was measured at 750 nm. The standard curve was prepared using gallic acid solutions with concentrations of 6.25 to 100 μg/ml. The samples were measured in triplicate.

### Bacterial Culture

The bacterial strain used in this study was *S. mutans* strain ATCC25175. These bacteria were obtained from the Department of Medical Sciences, Ministry of Health, Thailand. They were cultured in Todd-Hewitt broth and agar (Difco, USA) and maintained in an incubator containing 5% carbon dioxide at 37°C.

### Determination of the minimum Inhibitory Concentration and minimum Bactericidal Concentration (MBC)

The minimum inhibitory concentration (MIC) was determined using a broth dilution method. Mangosteen extract was dissolved in 95% ethanol. Then, two-fold serial dilutions were performed in the culture medium. Chlorhexidine digluconate at 0.12% was used as a positive control and was serially diluted in a similar manner. Medium without extract served as a control for bacterial growth. Each well was inoculated with bacteria obtained during the logarithmic phase of growth. The initial density of the bacteria was approximately 106 to 108 colony forming units (CFU)/ml.^20^ After a 24-hour incubation, the MIC was recorded as the lowest concentration that limited the turbidity of the broth to <0.05 at the absorbance of 600 nm. Solvent controls were also included, although no significant effect on bacterial growth was observed at the highest concentration employed. All of the wells from the MIC experiments that showed no visible turbidity were serially diluted, and 10 μl was dropped onto agar plates for viable cell counting. The plates were incubated for 24 to 48 hours. The MBC was then recorded as the lowest concentration that killed at least 99.99% of the initial number of bacteria. All MIC and MBC experiments were repeated three times.

### Time-kill Kinetics

The time-kill kinetics was determined using the number of remaining viable bacteria at varying time points. After exposure to mangosteen extract at the MBC for the specified times, the samples were diluted at least 10-fold by phosphate buffer saline (PBS) to arrest antibacterial activity and to reduce carry-over. The suspensions were then transferred onto agar using the drop plate technique for viable cell counting. The bacterial broth without extract served as a control for bacterial growth at each time point. The time kill curve was plotted as the logarithm of the number of remaining viable bacteria (log_10_ CFU/ml) against time. The sensitivity limit for detection was 10 CFU/ml. All assays were performed three times.

### Antibacterial Effects of Apacaries Gel

The antibacterial properties of Apacaries gel were tested using the agar-well diffusion method. This method is based on the same principle as the agar diffusion assay. The only change was the application of the sample. *S. mutans* (OD = 0.1, 108 CFUs/ml)^21^ in Todd-Hewitt broth was spread on mitis-salivarius agar, to which bacitracin was added to inhibit other streptococci. Each agar plate was punched out with 5 wells with diameters of 6 mm using a pasture pipette. For the agar-well diffusion method, the reservoir of the tested materials was used instead of a paper disk. The samples were incubated at 37°C for 24 hours. The antibacterial effect was recorded as the size of the inhibition zone.

## RESULTS

### The Result of TLC Chromatography

**Fig. 1:**
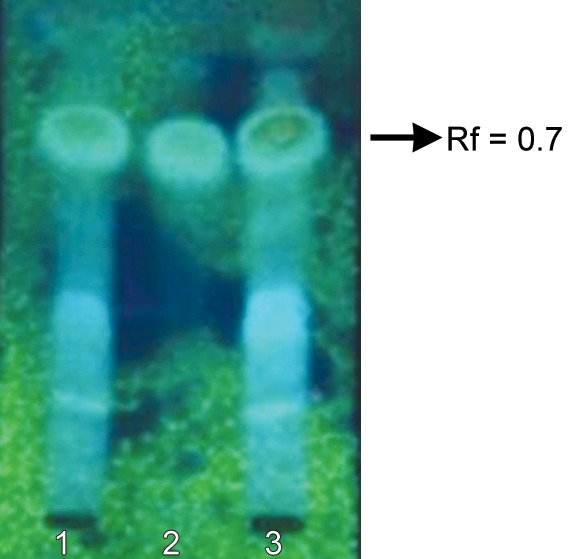
The TLC chromatogram of *G. mangostana* fruit pericarp extract under UV366 nm [absorbent: silica gel 60 GF254; solvent system: CHCl_3_-EtOAc-MeOH (8:1:0.5); the plate was sprayed with 10% sulfuric acid in ethanol and heated at 110°C for 10 minutes; lane 1 and 3 are mangosteen extract, lane 2 is standard a-mangostin at Rf = 0.7]

Mangosteen crude extracted was obtained from 95% EtOH maceration of mangosteen pericarp, and yielded 7.58% dry weight. The TLC chromatogram of mangosteen extract showed the presence of α-mangostin as a major component at Rf 0.7 (Fig. 1). The consisting of phenolic compounds was determined as the total phenolic content which was 25.88 mg GAE/100 mg crude extract.

### MIC and MBC of mangosteen Extract

The mangosteen pericarp extract was active against *S. mutans.* The MIC and MBC for ATCC25175 strain were 250 μg/ml and 1000 μg/ml respectively. The MIC and MBC of the crude extract were comparable to those of the control, which was bacterial broth, and of 0.12% chlorhexidine digluconate, an antiseptic commonly used in plaque control.

### Time-kill Kinetics

**Graph. 1:**
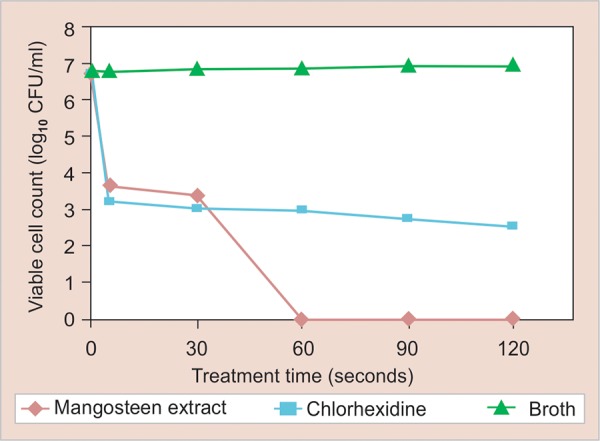
Time-kill curve for *S. mutans* ATCC25175 treated with mangosteen pericarp extract. The graph is plotted as the logarithm of the number of remaining viable cells (log_10_ CFU/ml) against time. The results are presented as the mean of three independent experiments

The time-kill curve was plotted as the logarithm of the number of remaining viable bacteria (log_10_ CFU/ml) against time as shown in Graph 1. The results show that applying 1 mg/ml of mangosteen extract can reduce *S. mutans* strain ATCC25175 by 50% within approximately 5 seconds with a lag time of 25 seconds. Afterward, the bacterial count rapidly drops to 0 at 60 seconds. Chlorhexidine, the positive control, showed a rapid drop in the first 5 seconds and remained stable for 2 minutes.

### Antibacterial Effects of Apacaries Gel

The results show that Apacaries gel with 1 mg/ml of mangosteen extract mixed in a 1:1.5 ratio with papain possessed the highest antibacterial property against *S. mutans* with an inhibition zone of 12.33 ± 0.29 mm (Table 1).

## DISCUSSION

The oral cavity contains many different bacterial species that interact with a human host to form a complex biofilm community. Of these bacteria, the mutans streptococci, in particular *S. mutans* are the most significant in the formation of dental caries.^22^ The amount of *S. mutans* in a biofilm has been correlated to the amount of caries risk.^23^ Therefore, the goal of many anticaries strategies is to reduce the levels of *S. mutans* in oral cavities. The current treatments for dental caries in human population, including water fluoridation and school-based program, are not sufficient to protect everyone. The scientific community has suggested the need for innovative work in a number of areas in cariology.^24^ Recent medical studies have suggested that the use of combinations of antibiotics allows a synergy of desirable pharmacological effects without necessarily increasing the undesirable side effects.^25^ However, continued exposure to antimicrobials promotes resistance development.^26^ Bacteria resistant to common oral antimicrobials, such as fluoride^27^ and xylitol,^28^ have also been found in oral cavities.

**Table 1:** **Table 1:** The inhibition zone of Apacaries gel and other materials against cariogenic *S. mutans*

*Materials*		*Diameter of inhibition zone* *(mean ± SD mm)*	
Mangosteen extract (0.1%) in gel preparation		8.83 ± 0.29	
Papain (59.94%) gel		7.83 ± 0.58	
Apacaries gel		12.33 ± 0.29	
SCMC gel base 3% w/w		0	
0.12% chlorhexidine digluconate		19.0	

SCMC: Sodium carboxymethylcellulose

New and better antimicrobial agents that are active against cariogenic bacteria without brittle taste are required, especially natural agents derived directly from plants. *Garcinia mangostana L*. was studied using repeated silica gel chromatography. α-mangostin was found to be a potent inhibitor of acid production by *S. mutans* and active against membrane enzymes, including F(H+)-ATPase and the phosphoenolpyruvate sugar phosphotransferase system. α-mangostin also inhibited the glycolytic enzymes aldo-lase, glyceraldehyde-3-phosphate dehydrogenase and lactic dehydrogenase’.^29^ *S. mutans* was inhibited by α-mangostin at concentrations of 12 and 120 μmol/l in a pH-dependent manner, with greater potency at lower pH values. Other targets for inhibition by α-mangostin include (i) malolactic fermentation, involved in alkali production from malate and (ii) NADH oxidase, the major respiratory enzyme in *S. mutans*.^29^ In addition, ‘papain gel has been utilized as a chemomechanical material for caries removal because of its ability to preserve underlying sound dentin’.^30^ In our study, we focused on the antimicrobial effects of a mango-steen extract and papain mixture in a gel preparation on *S. mutans*. The results from the TLC investigation showed that the mangosteen extract contains α-mangostin (*see* Fig. 1). The Folin-Ciocalteu colorimetric method used in the study revealed 25.88 μg/ml of total phenolic compound. We found that the mangosteen extract has a bactericidal effect against *S. mutans* strain ATCC25175, the MIC of which is 0.25 mg/ml. The time-kill kinetics curve showed that the application of 1 mg/ml of mangosteen extract can reduce *S. mutans* strain ATCC25175 by 50% within approximately 5 seconds with a lag time of 25 seconds. After this reduction, the bacterial count rapidly drops to zero within 60 seconds. Chlorhexidine, the positive control showed a rapid drop in the first 5 seconds and remained stable until 2 minutes (*see* Graph 1). The inhibition zone of the papain base gel is smaller than that of mangosteen extract. The papain mixture was isolated from the latex of the fruit and leaves of Carica papaya. Papain is a vegetable pepsin that cleaves bonds with the amino acids phenylalanine, tryptophan and tyrosine in proteins. Papain activity can hydrolyse the proteins in the outer portion of Gram-negative bacteria and, as a result, perturb the membrane permeability.^31^ The inhibition zone of mangosteen extract and papain mixture in gel preparation was larger than the zones for the separate components, indicating that papain and mangosteen have a synergistic effect on *S. mutans*.

## CONCLUSION

Our study showed that 1 mg/ml mangosteen extract mixed with papain in Apacaries gel can effectively inhibit *S. mutans* strain ATCC25175 within 2 minutes. The use of Apacaries gel is highly recommended for the treatment of patients seeking an alternative to conventional methods. Removal of carious tissue using Apacaries gel, which has antibacterial effects from mangosteen extract and papain, can be efficient, easy to perform and less destructive to dental tissues. The limitation of this study is our data could not be extrapolated to the clinical setting. Further studies should be focused to *in vivo* and clinical randomized control trials.

## References

[1] Alaluusua S, Renkonen OV (1983). Streptococcus mutans establishment and dental caries experience in children from 2 to 4 years old.. Europ J Oral Sci.

[2] Casamassimo PS, Thikkurissy S, Edelstein BL, Maiorini E (2009). Beyond the dmft: the human and economic cost of early child- hood caries.. J Am Dent Associ.

[3] Laitala M, Alanen P, Isokangas P, Söderling E, Pienihäkkinen K (2012). A cohort study on the association of early mutans streptococci colonisation and dental decay.. Car Res.

[4] Pandit I, Srivastava N, Gugnani N, Gupta M, Verma L (2007). Various methods of caries removal in children: a comparative clinical study.. J Indian Soc Pedodont Prevent Dentist.

[5] Banerjee A, Watson T, Kidd E (2000). Conservative dentistry: dentine caries excavation: a review of current clinical techniques.. Br Dent J.

[6] Maragakis GM, Hahn P, Hellwig E (2011). Chemomechanical caries removal: a comprehensive review of the literature.. Int Dent J.

[7] Maragakis G, Hahn P, Hellwig E (2001). Clinical evaluation of chemo-mechanical caries removal in primary molars and its acceptance by patients.. Car Res.

[8] Nakahara K, Kawabata S, Ono H, Ogura K, Tanaka T, Ooshima T (1993). Inhibitory effect of oolong tea polyphenols on glycosyl- transferase of mutans Streptococci.. Appl Environ Microbiol.

[9] Matsumoto M, Hamada S, Ooshima T (2003). Molecular analysis of the inhibitory effects of oolong tea polyphenols on glucan-binding domain of recombinant glucosyltransferases from Streptococcus mutans MT8148.. FEMS Microbiol Lett.

[10] Duarte S, Gregoire S, Singh AP, Vorsa N, Schaich K, Bowen WH (2006). Inhibitory effects of cranberry polyphenols on forma- tion and acidogenicity of Streptococcus mutans biofilms.. FEMS Microbiol Lett.

[11] Gregoire S, Singh AP, Vorsa N, Koo H (2007). Influence of cran- berry phenolics on glucan synthesis by glucosyltransferases and Streptococcus mutans acidogenicity.. J Appl Microbiol.

[12] Thimothe J, Bonsi IA, Padilla-Zakour OI, Koo H (2007). Chemical characterization of red wine grape (vitis vinifera and vitis interspecific hybrids) and pomace phenolic extracts and their biological activity against Streptococcus mutans.. J Agric Food Chem.

[13] Sturr MG, Marquis RE (1992). Comparative acid tolerances and inhi- bitor sensitivities of isolated F-ATPases of oral lactic acid bacteria.. Appl Environ Microbiol.

[14] Xiao Y, Liu T, Zhan L, Zhou X (2000). The effects of tea polyphenols on the adherence of cariogenic bacterium to the salivary acquired pellicle in vitro.. Hua Xi Kou Qiang Yi Xue Za Zhi.

[15] Li JY, Zhan L, Barlow J, Lynch RJ, Zhou XD, Liu TJ (2004). Effect of tea polyphenol on the demineralization and remineralization of enamel in vitro.. Sichuan Da Xue Xue Bao Yi Xue Ban.

[16] Bussadori SK, Castro LC, Galvao AC (2005). Papain gel: a new chemo- mechanical caries removal agent.. J Clin Pediatr Dent.

[17] Bussadori SK, Guedes CC, Hermida Bruno ML, Ram D (2008). Chemo- mechanical removal of caries in an adolescent patient using a papain gel: case report.. J Clin Pediatr Dent.

[18] Pothitirat W, Gritsanapan W (2009). HPLC quantitative analysis method for the determination of α-mangostin in mangosteen fruit rind extract.. Thai J Agricultural Sci.

[19] Singleton VL, Rossi Jr JA (1965). Colorimetry of total phenolics with phosphomolybdic-phosphotungstic acid reagents.. Am J Enology Viticult.

[20] Jrah Harzallah H, Kouidhi B, Flamini G, Bakhrouf A, Mahjoub T (2011). Chemical composition, antimicrobial potential against cariogenic bacteria and cytotoxic activity of Tunisian Nigella sativa essential oil and thymoquinone.. Food Chem.

[21] Mattana C, Satorres S, Sosa A, Fusco M, Alcaréz L (2010). Antibacterial activity of extracts of Acacia aroma against methicillin-resistant and methicillin-sensitive Staphylococcus.. Brazilian J Microbiol.

[22] Lester K, Simmonds R, Zoocin A (2012). Lauricidin in combination reduce Streptococcus mutans growth in a multispecies biofilm.. Caries Res.

[23] Loesche WJ (1986). Role of Streptococcus mutans in human dental decay.. Microbiol Reviews.

[24] Vieira A, Modesto A, Ismail A, Watt R (2012). Summary of the IADR cariology research group symposium, Barcelona, Spain, July 2010: new directions in cariology research.. Caries Res.

[25] Steenbergen JN, Mohr JF, Thorne GM (2009). Effects of daptomycin in combination with other antimicrobial agents: a review of in vitro and animal model studies.. J Antimicrobial Chemotherapy.

[26] Wright GD, Sutherland AD (2007). New strategies for combating multidrug-resistant bacteria.. Trends in Molecular Medicine.

[27] Streckfuss JL, Perkins D, Horton IM, Brown LR, Dreizen S, Graves L (1980). Fluoride resistance and adherence of selected strains of Streptococcus mutans to smooth surfaces after exposure to fluoride.. J Dent Res.

[28] Trahan L, Söderling E, Drean MF, Chevrier MC, Isokangas P (1992). Effect of xylitol consumption on the plaque-saliva distri- bution of mutans streptococci and the occurrence and long-term survival of xylitol-resistant strains.. J Dent Res.

[29] Nguyen PT, Marquis RE (2011). Antimicrobial actions of α-mangostin against oral streptococci.. Canadian J Microbiol.

[30] Bertassoni L, Marshall G (2009). Papain-gel degrades intact nonminera- lized type I collagen fibrils.. Scanning.

[31] Kim JH, Park WB, Park CY, Kim OK (1998). Antimicrobial activity of lysozyme and papain activited by EDTA and cysteine against Escherichia coli in culture medium and in aqueuos aloe vera extract.. Food Sci Biotechnol.

